# Schirmer test changes after 20 gauge and 23 gauge pars plana vitrectomy

**DOI:** 10.22336/rjo.2017.7

**Published:** 2017

**Authors:** Falavarjani Khalil Ghasemi, Yahya Shaheen, Moghaddam Arezoo Karimi, Hossein Aghaei, Mohammad Mehdi Parvaresh, Kashkouli Mohsen Bahmani, Hosein Farrokhi, Aghdam Kaveh Abri

**Affiliations:** * Eye Research Center, Rassoul Akram Hospital, Iran University of Medical Sciences, Tehran, Iran

**Keywords:** 20 gauge, 23 gauge, dry eye, pars plana vitrectomy, Schirmer test

## Abstract

**Objective:** To evaluate the short-term changes in Schirmer I test (ST) after pars plana vitrectomy and to compare the results between 23 gauge and 20 gauge vitrectomy surgeries.

**Methods:** 42 patients who underwent pars plana vitrectomy for posterior segment diseases were included in this prospective, non-randomized, comparative study. The choice of sclerotomy gauge was at the surgeons’ discretion. ST values were recorded before and at 1 and 3 months after vitrectomy.

**Results:** 20 patients in 23 gauge and 22 patients in 20-gauge group with a mean age of 59.9 ± 13.5 years were included. The mean preoperative ST values decreased significantly in both groups at 1 and 3 months after surgery (all P < 0.01). The ST values in the fellow eyes were the same, at baseline and during the follow up (P > 0.05). At 3 months visit, 15 eyes (35.7%) had abnormal ST measurements. There was no statistically significant difference in the changes in the ST measurements between the two groups at one month (P = 0.7), however, 3 months after surgery, the mean decrease in the ST measurements was significantly higher in the 20 gauge group (P = 0.03). At 3 months, 4 eyes in the 23 gauge group (20%) and 11 eyes in the 20 gauge group (50%) had abnormal ST measurements (P = 0.05).

**Conclusions:** Although both 20 and 23-gauge vitrectomy decrease the ST measurements postoperatively, the value is less affected by the 23-gauge vitrectomy.

## Introduction

Dry eye is a common condition, with an increased prevalence in the elderly and in patients with different systemic diseases including autoimmune diseases and diabetes mellitus [**1**,**2**]. Previous studies have shown that surgical trauma to the external ocular surface results in significant changes in the tear film characteristics, leading to the postoperative dry eye [**3**-**6**]. The changes are especially prominent in patients with preoperative signs of dry eye [**7**]. The effect of corneal refractive surgery and cataract surgery on the tear film is well understood. However, limited studies have reported ocular surface changes after pars plana vitrectomy [**8**-**11**]. Moreover, the effect of transconjunctival small gauge vitrectomy, which is associated with less manipulation of the ocular surface on the tear film and the incidence of dry eye after surgery, remains unclear [**12**]. 

The diagnosis of dry eye is based on the clinical features and some diagnostic tests such as tear break-up time, tear meniscus height, and rose Bengal staining. The assessment of tear production by Schirmer I test (ST) is the most common test in the evaluation of dry eye [**13**]. It measures the basal tear secretion and the function of the main lacrimal gland. In a literature search using the Pubmed database, no study reporting the ST measurements after pars plana vitrectomy could be found. 

The aim of this study was to evaluate the short-term effect of pars plana vitrectomy on the ST measurements in patients with no preoperative dry eye. Also, the ST changes after conventional 20 gauge versus microincision 23 gauge vitrectomy surgery were compared. 

## Methods

42 patients who underwent pars plana vitrectomy for various posterior segment diseases were included in this prospective, non-randomized case series study, from January 2012 to September 2013, and the Eye Research Center Ethics Committee approval and consent form from the subjects were obtained. Patients with clinical signs of dry eye, including preoperative ST values < 10 mm in either vitrectomy or fellow eyes, previous intraocular surgery, except for phacoemulsification more than 6 months before, known ocular surface disease (e.g. cicatricial conditions), acute inflammatory and/ or infectious ocular problems, and patients with a history of systemic autoimmune diseases (e.g. rheumatoid arthritis, systemic lupus erythematosus, Sjӧgren’s syndrome), were excluded. Also, patients who needed repeated surgery during the first 3 months after vitrectomy were excluded. 

The ST value without anesthesia was determined by measuring the length of the wetted part of the standardized filter paper strip (Whatman no. 41) at 5 minutes after inserting one end of the paper into the lateral side of the lower conjunctival fornix. The patients were instructed to keep the eyes closed during the test. Instillation of eye drops and extraocular surface manipulations were avoided at least 1 hour before the measurements. ST measurements were performed in both eyes before, and at 1 and 3 months after surgery. A ST measurement of < 10 mm was considered abnormal. 

All surgeries were performed by one surgeon (KGF). Standard 3-port pars plana vitrectomy was performed. The choice of the gauge depended on the surgeons’ decision. Generally, 20-gauge vitrectomy was selected in eyes with severe proliferative vitreoretinopathy or proliferative diabetic retinopathy, so that an extensive scleral depression or a circumferential buckle was necessary. In 20-gauge surgery, limited 30 and 90-degree limbal peritomies were performed at the superonasal and temporal part of the globe, respectively. A 360-degree peritomy was performed in eyes with forniceal shortening and when a circumferential buckle was needed. At the end of the surgery, the sclerotomy and conjunctiva were closed separately, by using 7-0 Vicryl sutures. In 23-gauge surgery, the trocars were inserted transconjunctival and no peritomy was performed. If there was a continuous leakage from the sclerotomy site at the end of the surgery and after removing the trocars, a 7-0 transconjunctival suture was used for closure. Subtenon injection of triamcinolone acetonide was performed for both groups at the conclusion of the surgery. Postoperative medication was similar between the two groups. Topical antibiotic eye drops were administered 4 times daily for 4 weeks and topical betamethasone eye drops were prescribed at every 2 hours for 1 week and then 4 times daily for the next 3 weeks. The Vicryl sutures were absorbed or removed within one month after surgery. 

Data were entered by using SPSS software (version 15, Chicago IL). Paired t-test, independent sample t-test, Fisher’s exact test, and Chi square test were used for statistical analysis and a P value of less than 0.05 was considered statistically significant. 

## Results

Forty-two patients including twenty women and twenty-two men, with a mean age of 59.9 ± 13.5 years, were included. The surgical indications were complications of diabetic retinopathy, rhegmatogenous retinal detachment, vitreous hemorrhage due to retinal vascular occlusion, macular hole, and macular pucker. The ST measurements at baseline were statistically the same for men and women (15.5 ± 4.4 and 18.1 ± 6.5, respectively, P = 0.1), and when eyes with diabetic retinopathy complications were compared with other surgical indications (18.5 ± 6.3 and 15.5 ± 4.2, respectively, P = 0.07). The baseline mean ST value was statistically similar between the vitrectomy eyes and fellow eyes (16.7 ± 5.6 and 14.9 ± 1.3 mm, respectively, P = 0.1).

The mean preoperative ST value of 16.7 ± 5.6 mm decreased to 11.9 ± 4.9 mm and 11.9 ± 4.8 mm at 1 and 3 months after surgery, respectively (both P < 0.001), in the operated eyes. The ST values were the same at baseline and during the follow up (P > 0.05), in the fellow eyes. The mean decrease in the ST values at 1 and 3 months (5.01 ± 5.5 and 4.7 ± 4.2 mm, respectively) were significantly different from the fellow eyes (1.1 ± 3.3 and 0.2 ± 2.9 mm, respectively, both P < 0.001). No significant difference was found in one and 3 months changes in the ST measurements between the eyes operated for diabetic retinopathy complications and those operated for other indications (P = 0.4 and P = 0.5, respectively). At 3 months visit, 15 eyes (35.7%) had abnormal ST measurements; however, all ST measurements in the fellow eyes were higher than 10 mm. 

Baseline characteristics and results of ST measurements in 20 and 23 gauge groups are summarized in **Table 1**. The mean ST values were decreased significantly at 1 and 3 months after surgery (all P < 0.01), in both groups. There was no statistically significant difference in the changes in ST measurements between the two groups at one month (P = 0.7). However, the mean decrease in the ST measurements was significantly higher in 20 gauge group at 3 months (P = 0.03). At 3 months, 4 eyes in the 23 gauge group (20%) and 11 eyes in the 20 gauge group (50%) had abnormal ST measurements (P = 0.05).

**Table 1:** Demographics and Schirmer I test results in 20 and 23 gauge vitrectomy groups

	20 gauge	23 gauge	P value
Number of patients	22	20	
Age (year)	58.1 ± 14.4	62.6 ± 12.3	0.4**
Gender (Male/ Female)	9/ 13	11/ 9	0.4†
Surgical indication (Complications of diabetic retinopathy/ other indications)	13/ 9	6/ 14	0.06 †
Baseline Schirmer test (mm)	17.6 ± 6.2	15.7 ± 4.8	0.3**
One month Schirmer test (mm)	13.2 ± 5.8	10.3 ± 2.9	0.06**
Three months Schirmer test (mm)	11.5 ± 5.7	12.4 ± 3.7	0.6**
Schirmer test measurement changes at 1 month (mm)*	5.2 ± 4.1	4.6 ± 6.7	0.7**
Schirmer test measurement changes at 3 months (mm)*	6.1 ± 4.6	3.3 ± 3.3	0.03**
Eyes with Schirmer test value < 10 mm at 3 months	4 (20%)	11 (50%)	0.05‡
* Compared to the baseline			
** Independent sample t-test			
† Chi square test			
‡ Fisher’s exact test			

## Discussion

Several mechanisms have been described for the tear film changes after intraocular surgery. Destruction of goblet cells, decrease in corneal sensation, and decrease in lacrimal gland secretion, alteration of the tear cytokines and disruption of corneal epithelium are amongst the reasons proposed to be responsible for postoperative dry eye [**3**,**5**,**8**,**14**]. 

Preoperative patient and ocular characteristics have been reported to affect the development of dry eye after surgery. These include age, sex, presence of dry eye and history of diabetic retinopathy [**2**,**7**,**9**]. Intraoperative factors such as the amount of exposure to microscope light, surgical trauma to the corneal epithelium, conjunctiva, eyelids, and lacrimal glands may cause significant changes in tear film [**2**,**5**,**7**,**9**,**14**,**15**]. Also, after surgery, some factors including topical eye drops and the presence of sutures may aggravate the tear dysfunction [**3**,**4**]. The patients were carefully selected to have a normal ST, being free of dry eye at baseline, to reduce the confounding effect of these variables. 

ST measurements were performed with eyes closed. Closing the eyes during ST results in less blinking, reducing the role of the lid margins and eyelashes in stimulating tear secretion and eliminating the influence of external factors such as temperature, evaporation, and humidity, and may help maintain more stable and uniform conditions [**13**]. Also, ST was performed on the fellow eyes and showed that the mean ST measurements remained the same after surgery. This showed that the effect of extraocular factors was negligible on ST measurements. Our results showed that the ST values significantly decreased after pars plana vitrectomy in both groups. Considering less than 10 mm of ST as abnormal [**16**], more than one third of this series had an abnormal ST at 3 months after surgery. 

Previous studies have reported higher rates of dry eye and corneal epitheliopathy after intraocular surgery in patients with diabetic retinopathy [**9**,**17**,**18**]. We did not observe any statistically significant difference between the eyes operated for the complications of diabetic retinopathy and others. 

Sutureless microincision vitrectomy has rapidly been replaced by the conventional 20-gauge system. The advantages of microincision vitrectomy include decreased surgical time, early postoperative rehabilitation and less postoperative discomfort [**12**]. Our study showed that compared to 20-gauge vitrectomy, the ST values are less affected after 23-gauge surgery, and the proportion of eyes with abnormal ST values was higher in 20-gauge group. The size and structure of the incisions obviated the need for a separate peritomy and reduced the conjunctival manipulation. Although the suture closure of the leaking sclerotomies might sometimes be necessary, a small transconjunctival suture is usually enough. Also, since the retina pathology is usually less severe in 23 gauge vitrectomy, the depression necessary for the anterior vitreous dissection is usually less extensive in 23 gauge surgeries. Other surgical characteristics including operation time, circumferential buckling, and epithelial debridement might further explain the difference in the ST results.

Our study had several limitations. The follow up period was short and the time interval needed for the normalization of the ST values was not clear. The small sample size might explain the absence of statistically non-significant results observed in some analyses. We did not match the 20 and 23 gauge groups based on the conjunctival opening, and sclera depression and did not randomize the study population. Moreover, ST could be affected by several factors including environment, age, and gender. However, to the best of our knowledge, this is the first study reporting the ST measurement changes after 20 and 23 gauge vitrectomy surgeries. Our results showed significant tear dysfunction after vitrectomy, which was more prominent in 20-gauge surgery. Future randomized studies with larger sample size are needed to confirm our results. 
